# Acetylcholine and an acetylcholinesterase inhibitor neostigmine can aggravate tularemia progress in BALB/c mice

**DOI:** 10.2478/v10102-012-0004-7

**Published:** 2012-03

**Authors:** Miroslav Pohanka, Oto Pavlis, Hana Svobodova, Jiri Pikula

**Affiliations:** 1 Faculty of Military Health Sciences, University of Defense, Hradec Kralove, Czech Republic; 2 Centre of Biological Defense, Techonin, Czech Republic; 3 Faculty of Veterinary Hygiene and Ecology, University of Veterinary and Pharmaceutical Sciences Brno, Czech Republic

**Keywords:** *Francisella tularensis*, intracellular pathogen, immunomodulation, cholinergic system, parasympathicus, cholinergic anti-inflammatory pathway

## Abstract

The present experiment was aimed at assessing the application of neostigmine, an acetylcholinesterase (AChE) pseudo-irreversible inhibitor with poor penetration through the hematoencephalitic barrier, and the neurotransmitter acetylcholine (ACh). The experiment was done to evaluate their ability to modulate an infectious disease: tularemia. Mice infected with *Franciselle tularensis* and exposed to either ACh or neostigmine had a higher mortality and spleen bacterial burden when compared to infected mice exposed to saline solution only. The activated cholinergic anti-inflammatory pathway suppressed pathways necessary for tularemia resolution. Administration of AChE inhibitors to the individuals suffering from tularemia is contra-indicatory. Drugs based on AChE inhibition should be restricted when tularemia or disease with a similar pathogenesis is suspected.

## Introduction

The immunomodulatory role of acetylcholine (ACh) has been considered for a long time (Kaufer *et al.*, 2003). The connection of the nervus vagus to the cholinergic anti-inflammatory pathway (CAP) as a specific regulation of the innate immune system by the parasympaticus was first recognized by Tracey and co-workers (Tracey, 2002). CAP was first referd to as reflex-like anti-inflammatory pathway, then the term CAP was introduced. The pathway is based on α7 nicotine acetylcholine receptor (nAChR) on the surface of macrophages and ACh is released into the blood system from the nervus vagus termination (Tracey, 2009). In the macrophages, the activated CAP inhibits the production of tumor necrosis factor alpha (TNF-α) and other pro-inflammatory intermediates (Wang *et al.*, 2003).

*Francisella tularensis* (Ft) is gram-negative bacterium causing the disease known as tularemia. It is considered one of the most important biological warfare agents. Ft is an intracellular pathogen preferably proliferating in macrophage phagosome (Anthony *et al.*, 1991). In the initial phase, interferon gamma (IFN-γ) and TNF-α are necessary for the resolution of tularemia (Fortier *et al.*, 1994). A major role of immune control of tularemia is based on activation of CD4+ and CD8+ T lymphocytes (Fulop *et al.*, 2001, Bosio, 2011). T lymphocyte receptor αβ knockout mice are sensitive to tularemia and can succumb more easily (Yee *et al.*, 1996).

The present experiment was focused on stimulation of CAP during the progression of tularemia infection. CAP is hypothesized to be able to modify tularemia progression on administration of either ACh as direct stimulator of nAChR or of the reversible acetylcholinesterase (AChE) inhibitor neostigmine, which protects endogenous ACh from splitting by blood AChE. In the final impact, both ACh and neostigmine could over-stimulate CAP localized in blood system without affecting of central nervous system (CNS), due to poor penetration through the blood-brain barrier (Yamamoto *et al.*, 1995). The achieved data relate to CAP and assess its practical impact.

## Material and methods

### Bacterium

*Francisella tularensis* LVS (ATCC 29684) was cultivated on McLeod agar supplemented with bovine hemoglobin and Iso VitaleX (Becton-Dickinson, San Jose, CA, USA) and processed as described previously (Pohanka *et al.*, 2007). Cells were harvested after two days of cultivation and were washed by saline solution using centrifugation 2,000×g for 10 minutes. Concentration of fresh cells was estimated using a cell density meter (WPA, Cambridge, UK) and confirmed by a cultivation test.

### Animals and tissues processing

Fiftysix three-month old female BALB/c mice (BioTest, Konarovice, Czech Republic) were divided into 7 groups. Each group contsisted of 8 animals. The mice were kept in an air conditioned room with steady temperature (22±2°C) and humidity (50±10%). The artificial light was adjusted at periods from 7 a.m to 7 p.m. The experiment was carried out within the vivarium of the Centre of Biological Defence in Techonin (Czech Republic) and was approved by the central ethical committee (Ministry of Defense, Czech Republic). Food and water was supplied *ad libitum* during the whole experiment. In the beginning of the experiment, the mice were eight weeks old and weighed on average 20 g. *F. tularensis* LVS was suspended in saline solution and adjusted to 10^5^ colony forming units (CFU)/ml. Ft LVS as well as neostigmine and ACh (Sigma-Aldrich; St.Louis, MO, USA) were suspended in saline solution prior to application. The groups were as follows:

- 100 μl of Ft suspension; 100 μl of saline solution
- 100 μl of Ft suspension; 100 μl of neostigmine 40 mg/l, dose 0.2 mg/kg body weight
- 100 μl of Ft suspension; 100 μl of acetylcholine 60 mg/l, dose 0.3 mg/kg body weight
- 100 μl saline solution; 100 μl of neostigmine 40 mg/l, dose 0.2 mg/kg body weight
- 100 μl of saline solution; 100 μl of acetylcholine 60 mg/l, dose 0.3 mg/kg body weight
- 100 μl of saline solution for two administrations.

The solutions were administered subcutaneously in the area of the pelvic limb. Neostigmine, ACh and saline solution were applied one hour after Ft suspension. Tularemia was confirmed in all infected animals by the appearance of typical disease signs.

After five days, mice were sacrificed under CO_2_ anesthesia. Spleens were collected and homogenized for cultivation in order to estimate the bacterial burden. The mortality experiment was carried out in the same way as given above. The dose of Ft was 100 μl 10^8^ CFU/ml. All other parameters were unchanged.

## Statistical analysis

Origin 8 (OriginLab Corporation, Northampton, MA, USA) was used for data processing throughout the experiments and performed for descriptive as well as inferential statistics. Significance of differences between the groups tested was estimated using one-way analysis of variance with Tukey's test. The significance was recalculated for two probability levels *p=*0.05 as well as *p=*0.01 for the group size n=8.

## Results

The bacterial burden of the spleen was assayed immediately after the animals had been sacrificed. In compliance with expectations, the animals that were not infected with tularemia had no positive cultivation proof. The animals infected with tularemia had on average content 5.50×10^4^ Ft CFU per spleen. Animals infected and simultaneously exposed to ACh or neostigmine had significantly (*p≤*0.01) increased Ft levels. The spleens from animals exposed to neostigmine had on average of 1.38×10^5^ CFU. The highest Ft content was found after ACh administration: 3.95×10^5^ CFU. The data are shown in Table 1.

**Table 1:** Bacterial burden in spleen of mice exposed to tularemia (Ft), tularemia with neostigmine (Ft + neo), and tularemia with acetylcholine (Ft + ACh).

Bacterial burden ± S.D. (CFU)		
Ft	Ft + neo	Ft + ACh
(5.50±3.83)×10^4^	(1.38±0.52)×10^5 **^	(3.95±1.33)×10^5 **^

Significance at *p=*0.01 against the first (Ft) group is indicated by the two asterisks.

**Figure 1:**
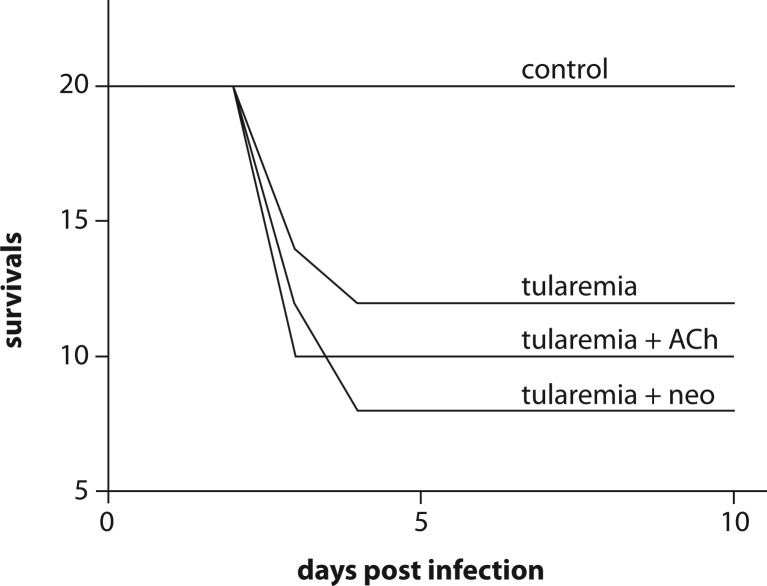
mortality after tularemia infection and acetylcholine (ACh) or neostigmine (neo) administration.

The mortality test compared the impact of the compounds tested on survival of tularemia infected animals (Figure 1) No mortality was observed in mice treated only with saline solution (control), ACh, or neostigmine. Infection with Ft caused 40% mortality. Co-application of ACh resulted in 50% mortality (comparison to Ft group: Chi Square 0.833; df=1; *p=*0.361) and of neostigmine in 60% mortality (Chi Square 3.33; df=1; *p=*0.067). The observed mortality occurred from the third to fourth day post infection. No mortality occurred before and after that time.

## Discussion

The multiple factors investigated during the experiments pointed to some interesting processes accompanying tularemia and/or CAP stimulation. The pyroptosis cell-death bears signs of necrosis and pro-inflammatory reaction (Bergbaken & Cookson, 2009; Kepp *et al.*, 2010). The stress markers arising during tularemia were also described in previous experiments (Pohanka *et al.*, 2009; Bandouchova *et al.*, 2009a). Generally, activation of CAP is expected to ameliorate inflammation induced pathogeneses as the direct impact on tissues can be diminished. On the other hand, activation of CAP can reduce the ability of the immune system to fight the pathogen.

Both ACh and neostigmine were administered in safe doses. The dose of ACh corrresponded to the mammalian physiological level (Fujii *et al.,* 1997). The neostigmine dose was correlated to the common medium amount per kilogram used in human medicine (Kopman and Eikermann, 2009). The most significant impact of neostigmine as well as of ACh was recognized in the bacterial burden and tularemia induced mortality. The bacterial burden was significantly increased in the spleen both after neostigmine and ACh administration. The bacterial burden corresponds to the organism's sensitivity to tularemia (Bandouchova *et al.*, 2009b) and an elevated bacterial burden should deductively correspond to higher mortality. In full agreement with the hypothesis, increased mortality was found after ACh as well as neostigmine administration.

CAP is linked to suppression of inflammation. When it is stimulated, the inflammation signaled by C-reactive protein and TNF-α is decreased (Jae *et al.*, 2009). The hypothesis set in the experiment was based on the question whether administration of compounds directly stimulating nAChR, Ach in the presaent study, or compounds inhibiting AChE, represented by neostigmine, and thus elevating endogenous blood ACh level, could modulate tularemia progression. Neostigmine was chosen on purpose for the fact that it does not cross the blood-brain barrier (Yamamoto *et al.*, 1995). The effect was not clearly comprehensible as parasympathetic rather than sympathetic response should be expected due to acetylcholine-evoked relaxation (Sotnikova *et al.*, 2009). The drug for Alzheimer′s disease treatment, Donepazil, has the same effect as it reduces stress and increases the ACh level even during exposition to bacterial liopolysaccharide (Tyagi *et al.*, 2010). On the other hand, the recognized oxidative stress after ACh administration could be triggered by another mechanism, not involved in ACh regulative function.

Stimulation of CAP and suppression of related cytokines production is well expected (Rosas-Ballina *et al.*, 2009). The primary question was to assess whether tularemia can be influenced by stimulation of CAP. The response was positive. Mortality and bacterial burden confirmed that stimulation of CAP in the first stage of tularemia is a negative process as the activated CAP does not allow simple activation of macrophages. The explanation is in full compliance with experiments of other authors. IL-1β deficient mice have a lower probability to survive tularemia (Fernandes-Alnemri *et al.*, 2010). Cytokines such as IFN-γ and TNF-α as well as T_H1_-lymphocytes are necessary to resolve the disease (Elkins *et al.*, 2009; Lin *et al.*, 2009). On the other hand, Ft needs to survive in macrophages and proliferate there in the phagosome (Bell *et al.*, 2010). Thus activated CAP could suppress pathways necessary for tularemia resolution. Administration of AChE inhibitors to individuals suffering from tularemia is contraindicatory. Drugs based on AChE should be restricted when tularemia is suspected. On the contraty, inhibition of CAP can be hypothesized as an appropriate approach since a positive effect can be expected.

## Conclusions

CAP seems to be a relevant regulatory tool influencing the immune system. Unsubstantial stimulation of CAP can deteriorate proliferation of the intracellular pathogen *F. tularensis*. Drugs based on AChE inhibition or nAChR stimulation are not applicable to individuals suspected to suffer from tularemia. Their impact is significantly contraindicatory. On the other hand, blockade of the nAChR is hypothesized as a suitable tool to be assessed in further experiments regarding tularemia treatment.
